# Effects of Antirejection Drugs on Innate Immune Cells After Kidney Transplantation

**DOI:** 10.3389/fimmu.2019.02978

**Published:** 2019-12-19

**Authors:** Gianluigi Zaza, Jeremy Leventhal, Lorenzo Signorini, Giovanni Gambaro, Paolo Cravedi

**Affiliations:** ^1^Renal Unit, Department of Medicine, University-Hospital of Verona, Verona, Italy; ^2^Department of Medicine, Icahn School of Medicine at Mount Sinai, New York, NY, United States

**Keywords:** innate immunity, kidney transplantation, calcineurin inhibitors, mTOR-inhibitors, mycophenolate mofetil, glucocorticoids

## Abstract

Over the last decades, our understanding of adaptive immune responses to solid organ transplantation increased considerably and allowed development of immunosuppressive drugs targeting key alloreactive T cells mechanism. As a result, rates of acute rejection dropped and short-term graft survival improved significantly. However, long-term outcomes are still disappointing. Recently, increasing evidence supports that innate immune responses plays roles in allograft rejection and represents a valuable target to further improve long-term allograft survival. Innate immune cells are activated by molecules with stereotypical motifs produced during injury (i.e., damage-associated molecular patterns, DAMPS) or infection (i.e., pathogen-associated molecular patterns, PAMPs). Activated innate immune cells can exert direct pro- and anti-inflammatory effects, while also priming adaptive immune responses. These cells are activated after transplantation by multiple stimuli, including ischemia-reperfusion injury, rejection, and infections. Data from animal models of graft rejection, show that inhibition of innate immunity promotes development of tolerance. Therefore, understanding mechanisms of innate immunity is important to improve graft outcomes. This review discusses effects of currently used immunosuppressive agents on innate immune responses in kidney transplantation.

## Introduction

For many years, strategies to prevent allograft rejection have focused purely on preventing adaptive immunity. Recent evidence has increasingly indicated that pure focus on T and B cells is not sufficient to improve long-term renal transplant outcomes (1–3). Innate immune cells (e.g., dendritic cells, monocytes, macrophages, neutrophils, NK cells), via numerous mechanisms, play an important role in all major immunological events following kidney transplantation (4). During the peri-transplant period, innate immunity is activated by donor brain death, ischemia-reperfusion injury, immunosuppression non-adherence, and infections—all of which increase risk for acute rejection (4–6). Late post-transplant innate immune cells produce an inflammatory microenvironment either in response to ongoing adaptive immune responses (e.g., chronic antibody mediated rejection) or independently, that enhances chronic allograft damage (7).

Innate immune cells are activated by common mechanisms. Molecules with stereotypical motifs produced during injury (i.e., damage-associated molecular patterns, DAMPS) or infection (i.e., pathogen-associated molecular patterns, PAMPs) initiate a variety of inflammatory events, including diapedesis, inflammatory cytokine production, and cell death (8). These pattern recognition receptor (PRR)-mediated inflammatory responses are necessary for microbial clearance. However, occurring post-transplant and resulting from release of endogenous PRR ligands, so-called “sterile inflammation,” they can lead to severe and often irreversible graft tissue damage and fibrosis (8, 9). Importantly, these phenomena provide a link to adaptive immune responses through induced costimulatory molecule expression and cytokine-mediated “help.”

Herein, we will review relevant literature regarding the impact of the main immunosuppressive agents employed in the maintenance phase of kidney transplantation (calcineurin inhibitors, mycophenolate mofetil/mycophenolic acid, corticosteroids, and mTOR inhibitors) on innate immune responses.

## Calcineurin Inhibitors

Calcineurin inhibitors (CNI), such as tacrolimus (TAC, FK-506) and cyclosporine A (CsA), still represent the mainstay of immunosuppression in kidney transplantation. Their dominant mechanism of action is the inhibition of Nuclear Factor of Activated T-cells (NFAT) phosphorylation, with consequent reduction of IL-2-mediated T lymphocyte activation and proliferation (10, 11). CNI may also inhibit cytokines secretion and effective antigen presentation in innate immune cells, reducing their T cell priming capacity (12, 13). All these activities are primarily involved in the pathogenesis of acute rejection and other transplant-associated comorbidities.

Evidence shows that CsA blocks NFAT binding to the inducible NO synthase (iNOS) promoter, causing a reduction of iNOS expression and nitrite production in macrophages (14, 15). CsA can also down-regulate the enzyme cyclooxygenase-2 (COX-2) in the kidney, which converts arachidonic acid into prostaglandin E2 (PGE2), an inflammatory mediator that modulates vascular permeability to expedite immune cell recruitment (16, 17).

CsA reduces the secretion of pro-inflammatory cytokines tumor necrosis factor (TNF)-α and IL-12 induced by LPS in human DCs (12, 18–20) and murine Langerhans cells (21, 22). On the other hand, CsA is able to increase the production of anti-inflammatory IL-10 in bone marrow derived DCs (BMDCs) and human blood-derived DCs induced by LPS (12, 18, 21). The inhibition of IL-12 and the induction of IL-10 mediate the ability of CsA to promote an anti-inflammatory phenotype on these DCs with consequent differential regulation of effector T cell subsets.

This effect could be enhanced in patients treated with anti-thymocyte globulins. As reported by Naujokat et al. (23), DCs are potential targets of anti-thymocyte globulins (ATGs). These agents can bind cell surface receptors on DCs and regulate some of their major immunological functions.

CNI have also an inhibitory effect on Toll-like receptors (TLRs) dependent activation of monocytes/macrophages. In monocytes/macrophages from liver transplant recipients, therapeutic concentrations of CsA impaired IL-6 production in response to TLR2 and TLR7/8 activation, and TNF-α synthesis due to TLR7/8 stimulation, more than TAC (24). In renal transplant recipients, a switch from CsA to TAC caused a large monocytes/macrophages response, measured as TNF-α, IL-1β, IL-6, and IL-10 production, further supporting the higher inhibitory effects of CsA on monocytes compared to TAC (25). The impairment of TLR function affects the risk of graft rejection, infection, and disease recurrence after transplantation, and the difference impact of CsA and TAC on monocytes should be considered in the choice of immunosuppressant therapy in order to improve the outcomes (24).

Moreover, although there are not conclusive findings regarding the effects of maintenance immunosuppressive drugs on innate immunity and their impact on ischemia reperfusion, the study of Yang et al. (26) suggested that CsA was ineffective to control innate immunity following ischemia reperfusion injury (IRI). In fact, this medication increased the infiltration of Endothelin-1 (ED-1+) (a specific rat monocyte/macrophage marker) cells in tubulointerstitium and periglomerular areas in rat kidneys undergoing IRI. Centrally, TAC had an opposite effect. A similar trend was seen for several inflammation cytokines (26).

CNI may also influence immune cells by affecting their mitochondrial function (27). In macrophages, mitochondrial cardiolipin, ROS, and DNA trigger IL-1β secretion by activating the NLRP3 inflammasome (28) and mitochondrial antiviral signaling protein (MAVS) oligomerization, inducing type I IFN production (29) and NFκB activation (30). CsA inhibits inflammasome activation preventing mitochondrial membrane permeability transition (MPT), thereby reducing inflammatory cytokine secretion (28).

In neutrophils CNI are able to inhibit ROS generation and the formation of Neutrophil Extracellular Traps (NET) (31), causing important functional or pathological effects. In Rag2^−/−^ mice, lacking B and T cells, CNI treatment induced a rapid development of *Candida albicans* infections, indicating that CsA impairs specific anti-fungal functions in innate immune cells (32). More specifically, mice lacking calcineurin activity in neutrophils were defective in the ability to kill *Candida albicans* indicating that CsA may directly influence neutrophil killing processes (32).

Currently, overall mortality due to fungal infections in transplant patients varies between 25 and 80%, with Candida and Cryptococcus species being the most commonly identified yeasts (33).

The higher doses of immunosuppressive medications in the first 6 months after transplantation are major causes of fungal infections. *Ex vivo* studies revealed that CsA damages human neutrophil clearance of *Aspergillus fumigatus* (another important cause of post-transplant opportunistic infections) (34), and that this effect is more evident in patients reaching high CNI trough levels. Inhibition of neutrophils activity by CNI may be, at least in part, responsible for increased risk of post-transplant fungal infections.

CNI do also affect NK cells in kidney transplant recipients (35). Zhang et al. have demonstrated that the expression levels of TNF-related apoptosis-inducing ligand (TRAIL) and FasL, potent apoptosis inducers, increase in NK cells at day 5 after transplantation, while their levels return to baseline on day 13 post-kidney transplantation (36). The authors also demonstrated that in supernatants generated from mixed lymphocytes culture (MLC) and on the surface of activated lymphocytes (particularly on NK cells) there was a significant increment of the expression of TRAIL and FasL. This condition was considerably reduced by adding CsA (500 ng/mL) at the beginning of MLC, an effect that could, at least in part, be implicated in the antirejection properties of CsA (36).

CsA inhibits the NK cells proliferation in a dose-dependent manner (37). Morteau et al. showed that *ex vivo* treatment of NK cells from healthy controls with CNI inhibits their degranulation and IFN-γ production. Similar functional impairment was observed in NK cells from CNI-treated patients. This could have dramatic effects on the NK cells capacity of killing transformed or virus-infected cells and producing pro-inflammatory cytokines and could, at least in part, explain the increased risk of opportunistic infections and tumors of CNI-treated patients (38).

## Mycophenolate Mofetil/Mycophenolic Acid

Currently, mycophenolate mofetil (MMF) and its active metabolite mycophenolic acid (MPA), are the most widely used drugs in transplantation (39, 40). MMF/MPA are considered specific anti-lymphocytes agents, since they reduce the *de novo* guanosine nucleotide synthesis by selectively inhibiting the inosine monophosphate dehydrogenase (IMPDH), mainly expressed by T- and B- cells (41, 42).

When exposed to MMF/MPA, monocytes show lower levels of pro-inflammatory cytokine IL-1β and altered polarization, with enhanced expression of surface markers (like CD163 and CD200R), generally associated with an anti-inflammatory function (M2 phenotype) (43). Additionally, MMF/MPA-exposed monocytes down-regulate several adhesion molecules, like ICAM-1, and display a weaker binding to cultured human umbilical vein endothelial cells (HUVEC) (44). Treating HUVECs alone with MMF/MPA does not reduce the adhesion of activated monocytes, reinforcing the idea of a direct effect of these compounds on monocytes (45).

In a mouse model of renal IRI, MMF down-regulated TLR4 expression on monocytes surface, along with plasma level of several cytokines (IL-6, MCP-1, and TNF-α). This resulted in milder kidney damage, as defined by creatinine levels and histological findings at 48 h after IRI (46).

MMF also reduces the LPS-induced expression of MHC-II on monocyte surface, suggesting a reduced activity as antigen presenting cells (44). In the presence of increasing MMF concentrations, human monocyte-derived dendritic cells (hMDDC) showed progressively less reactive phenotype. MMF treatment lowers the expression of costimulatory molecules (CD40, CD80, CD86), adhesion proteins (ICAM-1) and maturation markers (CD83, CD206), and decreases the synthesis of proinflammatory cytokines (TNF-α, IL-10, IL-12, IL-18) and alloreactive T-cells stimulation (47). When exposed to MMF, monocytes do also display higher rates of apoptosis (48).

MPA and MMF have similar effects on hMDDCs activation and maturation, but MMF reduces, instead of increasing, IL-10 synthesis. This may support the concept that MPA has stronger protolerogenic effects on monocytes compared to MMF (49). It is likely that these effects are independent of IMPDH inhibition.

MMF/MPA have also modulating effects on NK cell activity. Similarly to mTOR inhibitors, they significantly reduce the proliferation of these cells and inhibit the expression of CD56, associated with a highly reactive phenotype (50, 51). Accordingly, NK cells treated with these agents lose their cytotoxicity against K562 bone marrow target cells and reduced IFN-γ production upon target encounter (50, 51).

Taking together, these data suggest that MMF/MPA impair differentiation, maturation and function of various innate immunity cells, which may represent an additional mechanism of their immunosuppressive effects. Whether similar mechanisms are shared with azathioprine, an antiproliferative agents with similar antirejection effects (52), is unclear.

## Glucocorticoids

Glucocorticoids (GCs) are anti-inflammatory drugs employed in both induction and maintenance phase of immunosuppression after kidney transplantation. They inhibit the inflammatory response and leukocyte migration into inflamed tissues. They also accelerate resolution of inflammation by inhibiting vascular permeability and leukocyte distribution/trafficking, and by modulating death/survival and cellular differentiation programs (53, 54).

Until recently, it has been thought that the anti-inflammatory effects of GCs were linked to their ability to inhibit regulator of genes encoding pro-inflammatory cytokines (e.g., NFκB and AP-1) through a mechanism called “transrepression” (55). However, additional mechanisms include: (1) transcription of genes able to negatively interfere with the synthesis of inflammatory mediators; (2) repression of genes mediating immune cells activation; (3) synergism between glucocorticoid receptor and transcription factors leading to the induction of anti-inflammatory genes (56, 57).

Glucocorticoids may also have direct effects on innate immune cells. *In vitro*, methylprednisolone-treated monocytes show increased expression of anti-inflammatory cytokines, like IL-10, with concomitant down-regulation of TNF-α, IL-1β, and IL-12 (58–60). Furthermore, GC-treated monocytes show lower expression levels of CD80 in response to inflammatory stimuli, which impairs their antigen-presenting activity (61). *In vivo*, data from methylprednisolone-treated kidney transplant patients, show increased numbers of CD14^++^CD16^−^ (classical) and CD14^++^CD16^+^ (intermediate) monocytes, while the CD14^+^CD16^++^ (non-classical) population is declined compared to patients receiving CNI, MMF/MPA or mTOR inhibitor (62). This is consistent with recent observations showing a downregulation of TLR4 level on the surface of GC-treated monocytes. TLR4 is a pivotal element of the monocyte activation during sepsis, as well as in the acquired immune response to transplanted organs (63). GCs reduce the *in vitro* expression of TLR4 and the response to endotoxin in monocytes through the mediation of micro-RNA (MiR) 511-5p, a keystone in the anti-inflammatory effect of GCs (64).

GC affects also DC differentiation and maturation. In fact DC differentiated from human monocytes in presence of dexamethasone expressed lower levels of CD83 and CD86, lower APC function and a lower capacity to secrete TNF-α and IL-1β induced by CD40L and LPS than untreated cells (65, 66).

It is well-known that the administration of GCs induces neutrophilic leukocytosis, in particular by promoting neutrophil maturation and mobilization (67), an effect that is blocked by simultaneous inhibition of the L-selectin adhesion protein (68, 69).

The entire neutrophil activation process is also inhibited by GCs that reduce the expression of enzymes related to respiratory burst, such as NADPH oxidase, iNOS and COX-2 (70–73), as well as processes of chemotaxis, phagocytosis, and cytokines secretion (74, 75). In neutrophils, GCs simultaneously inhibit transcription factors related to pro and anti-inflammatory genes. The net effect is an increase in the expression of some receptors for interleukins and pro-inflammatory leukotrienes, such as IL1R1 and BLT1, (76–78), as well as a reduced sensitivity to apoptosis which increases neutrophils average life span (79).

NK are also sensitive to the effects of endogenous glucocorticoids under stress conditions, when steroids reduce NK cytolytic activity (80–82). Recent evidence shows that GCs can also induce the synthesis of pro-inflammatory cytokines through an epigenetic mechanism in NK cells. In particular, the expression of IL-6 and INF-γ is increased, along with a greater histone acetylation in the enhancer regions of these genes, which are thus more easily accessible to activating transcription factors (83, 84).

## The Mammalian Target of Rapamycin Inhibitors: Sirolimus and Everolimus

The mammalian target of rapamycin (mTOR) is part of 2 different complexes (mTORC1 and mTORC2) with diverse signaling networks. mTORC1 promotes anabolic cellular metabolism stimulating synthesis of proteins, lipids, and nucleotides and, at the same time, inhibits catabolic processes, such as lysosome biogenesis and autophagy. mTORC2 controls cell survival, cytoskeleton organization, lipogenesis, and gluconeogenesis (85). In organ transplantation mTOR inhibitors, Sirolimus, and Everolimus, exert their immunosuppressive functions by preferentially inhibiting mTORC1 (86) thereby ostensibly halting protein translation necessary for effector T cell proliferation. Additional experimental and clinical experience with mTOR inhibitors support that they exert effects on graft survival, both beneficial and detrimental, in part by acting on innate immune cells (87, 88). Via changes in antigen presentation and costimulatory molecules, cytokine production, and metabolic pathways, mTOR inhibitors produce extensive, and sometimes conflicting, effects on innate immune cells.

The mTOR network allows innate immune cell maturation and costimulatory molecule expression during inflammation (89). As might be predicted, treatment with mTOR inhibitor impairs DCs maturation after LPS stimulation by reducing translation, including that of MCH-II and costimulatory molecules (90). Rapamycin hampers functional and phenotypic maturation of DCs prompted by IL-4, LPS, or CD40 ligation (91–93) and impairs their ability to stimulate effector T cell proliferation. Similarly, DC development induced by fms-like tyrosine 3 kinase ligand (Flt3L), a powerful DC growth factor, is inhibited by rapamycin (93, 94). Accordingly, the DCs antigens uptake activity is impaired which further contributes to damaged allogeneic T lymphocytes stimulation (90, 95).

Conversely, mTOR inhibitors indirectly inhibit regulation of autophagy and promote this degradation with immunoregulatory capabilities. Importantly autophagy is a well-known contributor to both MHCII presentation and MHCI cross-presentation of exogenous peptides (96, 97). Increased antigen presentation increases the risk of activating adaptive immune responses and is an unintended and unwanted consequence of mTOR inhibitor use. In a murine liver transplant model, use of autophagy inhibitors improved graft and animal survival, although whether this was mediated by MHC presentation effects is unknown. Regardless, induction of innate immune cell autophagy is potential counterproductive side effect of mTOR inhibitor (98).

In immature DCs, mTOR inhibitors induce apoptosis by blocking the granulocyte-macrophage colony-stimulating factor (GM-CSF) signaling. Disruption in GM-CSF/PI3K/mTOR pathway produces a pro-apoptotic state, unbalancing anti- and pro-apoptotic mediators by reduction of the mitochondrial membrane potential (99). In mature DCs, PI3K/mTOR inhibition with increasing drug concentrations down-regulates progressively several pro-inflammatory cytokines of the monocytic/macrophagic repertoire, in parallel with the reduction of phosphorylated Akt and p706K levels (90, 100, 101). In addition, mTOR inhibitor causes apoptosis in both human monocyte-derived and CD34+-derived DCs, without any effect in macrophages or myeloid cell lines (102).

mTOR inhibitors inhibit NK cell inflammatory capabilities by inhibiting their cytokine expressing and cytotoxic function. In particular, rapamycin impaired growth of the CD56^bright^CD16^+/−^ NK cell subset (associated with enhanced cytokine production) without affecting the amount of CD56^dim^CD16^+^ cells subset (with more cytotoxic capacity). With regards to the cytotoxic subset, mTOR inhibitors prevented NK cell expression of NKG2A and NCR (51). Absence of receptor ligation (if present) by target cells induces NK cell cytotoxic activity against the target cell. Prevention of their expression by mTOR inhibitors, therefore, impairs NK cell cytotoxic functioning. Overall, these results demonstrate that, mTOR inhibitors have distinct deleterious effects in immune cells which may have important implications in transplantation (51).

An essential innate immune cell role involves production of cytokines. The mTOR inhibitors have pleiotropic effects that depend on the cells and circumstances studied. In LPS-stimulated human monocytes, mTOR inhibitors reduce several pro-inflammatory chemokines synthesis such as MCP-1, RANTES, IL-8, and MIP-1 (103). Fine-needle aspiration biopsy (FNAB) samples (containing mononuclear cells together with kidney parenchymal cells) obtained from kidney transplant recipients receiving sirolimus showed lower synthesis of many proinflammatory cytokines, including IL-6 and MCP-1, and higher production of TGF-β than samples from patients whose regimen contained MMF (104). Conversely, switch from a CNI based to a mTOR inhibitor-based regimen may worsen post-transplant inflammation. Gene expression profile on kidney samples showed the upregulation of pathways involved in production of NO, ROS, and IL-12 in macrophages and the activation of the adaptive immune response. Histological analysis confirmed a higher macrophages infiltration (105). Similarly, after shift from CsA to Sirolimus, the transcriptomic analysis on peripheral blood leucocytes showed a significant enrichment in pro-inflammatory pathways related to NFκB and specific transcripts for monocyte and NK cells (106). It is noteworthy that the concomitant administration of mTOR inhibitors and GCs seem to cause a state of innate immune cell hyper-responsiveness, as if GCs action is override by the inhibition of mTOR (107).

mTOR inhibitor modulation of innate immune cells may contribute to a pro-tolerogenic state in the early phases of transplantation. Sordi et al. (108) showed that sirolimus, at clinically relevant concentrations and in contrast to calcineurin inhibitors, enhances the expression of CCR7 on the surface of human and mouse derived DCs with consequent expedite migration of DCs into lymphoid tissue. This condition may promote the tolerogenic effect of mTOR inhibitors, because these immune cells may reach appropriate T cell areas in the lymphoid tissue (109). Recent evidence challenged long-held notions that immunological memory is a feature exclusively for adaptive immunity. Evidence in monocytes showed that beta-glucan (a fungal antigen) experienced monocytes developed epigenetic changes spurred by accumulation of a cholesterol intermediate, mevalonate (110). Epigenetic changes were dependent on activation of mTOR to induce necessary downstream metabolic and histone changes. Importantly, using a strategy that included innate immune targeting rapamycin loaded nanoparticles, Braza et al. prevented macrophage trained immunity and extended graft survival indefinitely. These findings lead to an intriguing possibility that short term myeloid-specific nanoimmunotherapy that targets mTOR inhibitor in post-transplant may extend graft survival by preventing trained immunity generation (111).

There are controversial data about the impact of the m-TOR inhibitors on the regulation of I/R injury-related innate immune system in kidney transplantation. Several authors have suggested that these drugs may impair recovery of kidney function (112–114) because of an anti-proliferative effects on tubular cells and an hyper-expression of several pro-inflammatory cytokines (e.g., IL1-β, IL-12, TNF-α) and an inhibition of the production of anti-inflammatory cytokines as IL-10. On the contrary, Macedo et al. have reported that m-TOR inhibitors may protect from innate immunity activation (115). In particular the inhibition of mTOR may induce resistance to phenotypic maturation of DCs induced by inflammation and may facilitate the production of regulatory tolerogenic DCs.

## Conclusions

In the past, great strides in allograft survival prolongation were attributed to successful suppression of adaptive immune responses (Table 1). A great body of literature, both clinical and basic science, attests to profound and diverse effects of modern immunosuppressive agents on innate immune cells. To make further progress improving transplant outcomes requires a more complete understanding of these effects and attempts to blunt current insufficiencies or vulnerabilities. As an example, clinical trials using monoclonal antibodies against innate immune receptors TLR2 (NCT01794663) and/or TLR4 (NCT01808469) to prevent delayed graft function and innate immune cell based therapies (including administration of regulatory macrophages and tolerogenic DCs) (116) may lead to new therapeutics that become standard of care to decrease the need for, or even completely replace, current immunosuppression regimens. These efforts to enlarge the post-transplant armamentarium by targeting innate immune cells will ideally lead to prolonged allograft function and minimized immunosuppression that extend allograft longevity without overly immunosuppressing and endangering the patient.

**Table 1:** Main effects of the immunosuppressive drugs on innate cells.

**Drug**	**Dendritic cells**	**Phagocytes**	**Natural Killer (NK)**
CNIs	Reduce LPS-induced secretion of pro-inflammatory cytokines TNF-α, IL-12 (12, 18–20).	Impair IL-6 and TNF-α production in response to TLR2 and TLR7/8 activation in **monocytes/macrophages** (24).	Reduce the expression levels of TNF-related apoptosis-inducing ligand (TRAIL) and FasL (36).
	Increase LPS-induced production of IL-10 in bone marrow derived DCs (BMDCs) and human blood-derived DCs (12, 18, 21).	Inhibit inflammasome activation preventing membrane permeability transition (MPT) in **monocytes/macrophages** (28).	Inhibit proliferation of NK cells in a dose-dependent manner (37).
	These effects may promote an anti-inflammatory phenotype on DCs that may lead to differential regulation of effector T cells subsets.	Inhibit **neutrophil's** reactive oxygen species generation and the formation of Neutrophil Extracellular Traps (NET) (31). This effect on neutrophil activity may be responsible for increased risk of post-transplant fungal infections.	Inhibit degranulation and IFN-γ production (38).
MMF/MPA	Lower the expression of costimulatory molecules (CD40, CD80, CD86), adhesion proteins (ICAM-1) and maturation markers (CD83, CD206) (47).	Inhibit IL-1β production and enhance the expression of surface markers of M2 phenotype (CD163 and CD200R) in **monocytes** (43).	Reduce proliferation of NK cells and inhibit the expression of CD56 (50, 51).
	Decrease the synthesis of proinflammatory cytokines (TNF-α, IL-10, IL-12, IL-18) (47).	Down-regulate adhesion molecules, like ICAM-1 in **monocytes** and inhibit their adhesion to endothelial cells (44).	Reduce cytotoxicity against K562 bone marrow target cells and IFN-γ production upon target encounter (50, 51).
	MMF reduces IL-10 synthesis (49).	Down-regulate TLR-4 expression on **monocytes** surface in a mouse model of Ischemia reperfusion injury resulting in milder kidney damage (46).	
		Reduce the LPS-induced expression of MHC-II on **monocyte** surface (44).	
		Induce apoptosis in **monocytes** (48).	
GCs	Reduce the production of TNF-α, IL-1β induced by CD40L and LPS (65, 66).	Increase expression of anti-inflammatory cytokines (IL-10) with concomitant down-regulation of TNF-α, IL-1β, IL-12 in **monocytes** (58–60).	Reduce NK cytolytic activity (80–82).
	Inhibit the LPS-induced up-regulation of costimulatory molecules (e.g., CD40, CD80, CD83, CD86, and MHC-II) (65, 66).	In **monocytes** GCs reduce the expression of CD80 in response to inflammatory stimuli which impairs their antigen-presenting activity (61).	Through an epigenetic mechanism GCs induce the synthesis of pro-inflammatory cytokines (83, 84).
	DC differentiated in the presence of GC are not able to induce the proliferation of allogeneic CD4 T cells (65, 66).	In kidney transplant patients, increase the number of CD14++CD16- and CD14++CD16+ **monocytes** while the CD14+CD16++ population is declined compared to patients receiving CNI, MMF/MPA or mTOR inhibitor (62).	
		Down-regulate TLR4 expression on the surface of **monocytes** and their response to endotoxin (64).	
		Inhibit activation process of **neutrophils** by reducing the expression of NADPH oxidase, iNOS, COX-2 (70–73).	
		Reduce chemotaxis, phagocytosis and cytokines secretion in **neutrophils** (74, 75).	
		Increase the expression of some receptors for interleukins and pro-inflammatory leukotrienes such as IL1R1 and BLT1 in **neutrophils** (76–78).	
		Reduce sensitivity to apoptosis which increases **neutrophils** average life span (79).	
mTOR inhibitors	Impair DC maturation after LPS stimulation by reducing translation, including that of MHC-II and costimulatory molecules (90).	In LPS-stimulated human **monocytes** reduce chemokines synthesis such as MCP-1, RANTES, IL-8, and MIP-1 (103).	Inhibit NK proliferation and cytotoxicity capacity (51).
	Prevent phenotypic and functional maturation induced by IL-4, LPS, or CD40 ligation (91–93).		
	Inhibit DC development induced by Flt3L (93).		
	Impair antigen uptake contributing to damage allogeneic T lymphocytes stimulation (95).	Induce the up-regulation of pathways involved in production of nitric oxide, reactive oxygen species and IL-12 in **macrophages** (105).	Inhibit the shift toward an overall NKG2A+KIR-NCR+ phenotype and maintain an overall NKG2A-KIR+NCR+/– (51).
	Disinhibit autophagy that contributes to both MHCII presentation and MHCI cross-presentation of exogenous peptides (96, 97).		
	Induce apoptosis in immature DC by blocking GM-CSF signaling (99).		
	Increase surface expression of chemokine receptor CCR7 promoting DC migration into lymphoid tissue (108).		

## Author Contributions

GZ, JL, and PC searched the literature and wrote the manuscript. LS contributed to the literature search and literature analysis. GG and PC revised the manuscript. All authors read and approved the final manuscript.

### Conflict of Interest

The authors declare that the research was conducted in the absence of any commercial or financial relationships that could be construed as a potential conflict of interest.
